# Loneliness and coping skill mediate the association between child maltreatment and depression for rural males and females

**DOI:** 10.1186/s12888-022-04056-1

**Published:** 2022-06-21

**Authors:** Meiqi Wang, Meixia Xu, Long Sun

**Affiliations:** 1 Centre for Health Management and Policy Research, School of Public Health, Cheeloo College of Medicine, Shandong University, 44 Wenhuaxi Road, Jinan, 250012 Shandong China; 2 National Health Commission of China, Key Lab of Health Economics and Policy Research (Shandong University), Jinan, 250012 Shandong China; 3 Shandong Women’s University, Jinan, 250300 China

**Keywords:** Child Maltreatment, Depression, Loneliness, Coping Skill, Mediation Models, Rural, China

## Abstract

**Background:**

Child maltreatment is a prevalent and notable problem in rural China, and the prevalence and severity of depression in rural areas are higher than the national norm. Several studies have found that loneliness and coping skills respectively mediated the relationship between child maltreatment and depression. However, few studies have examined the roles of loneliness and coping skills in child maltreatment and depression based on gender differences.

**Methods:**

All participants were from rural communities aged more than 18 years in Shandong province, and 879 valid samples (female:63.4%) ranging in age from 18 to 91 years old were analyzed. The Childhood Trauma Questionnaire-Short Form (CTQ-SF), the Center for Epidemiologic Studies-Depression (CES-D), the Simple Coping Style Questionnaire (SCSQ), and the Emotional and Social Loneliness Scale (ESLS) were used to evaluate child maltreatment, depression, coping skills and loneliness.

**Results:**

Child maltreatment was more common and severe in males than females (F = 3.99; *p* < 0.05). Loneliness and coping skills partially mediated the relationship between child maltreatment and depression in males, but loneliness fully mediated the relationship between child maltreatment and depression in females.

**Conclusion:**

In this study, males were more likely to experience child maltreatment. Child maltreatment and depression were correlated. We also found a mediating role of loneliness and coping skills for males and a mediating role of loneliness in females.

## Background

Child maltreatment is a public health problem worldwide [1, 2], and can influence victims’ mental health from the period of adolescence to adulthood to lifetime. There are five types of child maltreatment: physical abuse, emotional abuse, sexual abuse, physical neglect, and emotional neglect. In rural China, the estimated prevalence of any child maltreatment has remained at 66.3% [3]. Boys were more likely to be physically abused than girls, and girls were more likely to be neglected because of the parental gender-linked expectations for children [4]. A school-based study reported that 43.09%, 41.65%, and 42.18% of rural children experienced physical abuse, emotional abuse, and neglect, respectively [5]. Of the boys, 80.5% suffered abuse or neglect, while 75.1% girls experienced any abuse and neglect in regions with a moderate level of economic growth [6]. A systematic review showed that 26.6%, 19.6%, and 26.0% of children suffered physical abuse, emotional abuse, and neglect [7]. We discovered that the prevalence of child maltreatment in rural China was higher than the national norm in China. Consequently, child maltreatment in rural areas is a prevalent and notable issue.

Depression is also a major public health issue and a common disease among adolescents and geriatric patients in China [8, 9], which influences people’s physical and mental health and increases the risks of suicide and morbidity. A meta-analysis reported that 22.7% of geriatric patients had depressive symptoms, women were more likely to develop depressive symptoms than men, and rural populations developed depressive symptoms more easily than urban populations [10]. Among the rural elderly in China, 54.7% developed depressive symptoms [11]. The prevalence and severity of depression in rural areas were higher than average. Depressive disorders ranked 19^th^ up to 13^th^ as the leading cause of the global burden of disease (GBD) from 1990 to 2019 and ranked 4^th^ and 6^th^ for 10–24 years and 25–49 years, respectively [12].

Child maltreatment is a potent risk factor for internalizing problems, such as depression, anxiety and loneliness [13]; sufficient studies have shown that females are vulnerable to depression and experience child maltreatment compared with males who experience child maltreatment in China [3, 14]. Child maltreatment is also a risk factor for developing maladaptive coping skills [15]; people who do not have adaptive coping skills may find it difficult to confront stressful events and regulate emotional problems and are prone to depression. Previous studies have indicated that coping skill was associated with depression symptoms [16, 17]. Thus, coping skills are a positive factor in the prevention of depression.

Studies have found that child maltreatment was associated with loneliness, coping skills and depression [18, 19]. Child maltreatment was positively correlated with loneliness and depression and negatively correlated with coping skill. Several studies examined that loneliness and coping skills mediated the relationship between child maltreatment and depression [20, 21]. However, few studies have examined the roles of loneliness and coping skills in child maltreatment and depression based on gender differences. Exclusive research investigated the mediating effects of different coping styles on the relationship between childhood maltreatment and depressive symptoms among Chinese male and female undergraduates [22], but coping styles consisting of six dimensions differed from coping skills in this study. Other studies have reported the mediating effect of coping skills in young male or female adults [21, 23].

This study aimed to examine the roles of loneliness and coping skills in child maltreatment and depression among rural males and females in China to understand better the cognitive-affective mechanisms underlying child maltreatment and depression and improve the prevention and intervention processes for depression.

### Child maltreatment and depression

Maltreatment can negatively impact development by altering the developing neural system or disrupting other factors. Furthermore, maltreatment may exacerbate or express neuropsychiatric syndromes in individuals with genetic vulnerabilities (e.g., major depression) [24]. People who are exposed to maltreatment in childhood are at risk of having a range of poor mental outcomes, such as major depressive disorder (MDD), posttraumatic stress disorder (PTSD), and substance abuse [25–27]. Specifically, numerous studies have suggested that child maltreatment is associated with depression during childhood, adulthood, and the geriatric period [28–30]. A retrospective study indicated that patients with depression experienced more severe childhood maltreatment than healthy controls [31].

Other studies have shown that the number of child maltreatment is associated with increased depressive symptoms [32, 33]. Studies found that many elderly people in Brazil who reported cumulative maltreatment experiences were more likely to suffer from depression, but there was no impact on the severity of depression [34]. However, it is not clear whether the influences of child maltreatment on depression is similar or different according to sex. Some studies have indicated that women who experienced child maltreatment were more likely to experience depression than men [14, 35]. Few studies have illustrated the reverse results that the men who experienced maltreatment were likely to have depression [36]. Moreover, several studies found a similar effect of child maltreatment on depression for males and females [37, 38].

### Child maltreatment and loneliness

Loneliness has been characterized as a feeling of social isolation and separateness [39, 40]. People isolated from society find it difficult to build and maintain social connections and acquire social support; thus, they become lonely. The feeling of loneliness is more intense from middle age onward. One study estimated that twenty-eight percent of older Chinese people reported feeling lonely, and approximately seven percent reported often or always feeling lonely [41]. A growing body of literature indicates that loneliness is associated with physical and mental health and cognitive functions, such as depressive symptoms [42], mortality [41], systolic blood pressure [43], and impaired cognition (Alzheimer’s disease) [43].

Studies have shown that childhood trauma is positively correlated with loneliness [44, 45], meaning that individuals who have experienced childhood trauma are easier to lonely than those who have not. Most importantly, previous studies have pointed out a relationship between child maltreatment and loneliness [13, 46].

Converging evidence provides empirical support for other studies. One study reported that childhood maltreatment is non-negligible for loneliness in adulthood [47]. Women who had been maltreated were lonelier and had a more negative network orientation than non-abused women because they tended to isolate themselves socially [48]. Findings from several studies indicate that children exposed to abuse also experience loneliness and social isolation in their lives, preventing the development of developing adequate and efficient social skills [49, 50].

### Child maltreatment and coping skill

Coping skills represented the way in which individuals deal with stressful or negative experiences [51]. In maltreating families, maltreating parents often conceal emotional expressions, interact in hostile and aggressive ways, and rely on punitive interaction styles. Based on this, due to high levels of unpredictability in parent–child interactions and the home generally, maltreated children fail to model appropriate coping skills when they encounter with stress and try to control what happens to them, leading to a feeling of helplessness [18].

Previous studies have reported that child maltreatment is associated with coping skills [52, 53]; people who experienced maltreatment did not cope with stress or regulate emotion and had low coping skills. Child maltreatment plays a major role in adolescent well-being and coping [54].

### Loneliness as a mediator

People who have experienced child maltreatment are likely to withdraw from society; they are reluctant to contact others due to feelings of inferiority and distress, so they are unable to receive social supports or concerns and eventually become lonely. The association between loneliness and depressive symptoms appears to be stable across ages [42], A lonely person is more likely to be depressed than a normal person.

Studies have investigated the role of loneliness as a mediator in the relationship between childhood trauma and adult psychopathology and indicated both direct and mediational effects of social resources on adult depression symptoms in women with a history of child multi-type maltreatment [32]. Loneliness mediates the relationship between children abuse and six adult psychiatric disorders: depression, generalized anxiety disorder (GAD), mixed anxiety and depression (MAD), phobia, post-traumatic stress disorder (PTSD), and psychosis [20].

### Coping skills as a mediator

On the one hand, child maltreatment causes poor coping skills, making it harder for maltreated children to confront and deal with stress. Meanwhile, the relationship between stress and major depression has been ensured [55], and stress increases the likelihood of depression. However, a high level of coping skills mediates or decreases the impact of maltreatment on depression.

Compared to non-maltreated children, child maltreatment is associated with a decrease in the usage of coping skills, and low coping skills may exacerbate depression. Studies have shown that coping skills mediated the relationship between child maltreatment and internalizing and externalizing behaviors [15]. Other studies have also found that coping skills mediate and moderate the impact of maltreatment on depressive symptoms [23].

### Current study

This study aims to answer two research questions. First, we examined the role of loneliness and coping skills in the relationship between child maltreatment and depression. Second, we tested whether the mediation models are differed by gender.

## Methods

### Participants

This is a cross-sectional study was conducted in Shandong province, China. All the participants were from one county (Taierzhuang). Shandong Province is located in the east of China, with economic prosperity in both industry and agriculture [56]. Taierzhuang County is located in the south of Shandong, and there were 230 thousands rural people [57]. We used the random cluster sampling method, and all five towns in Taierzhuang County were selected to conduct the interviews. In each town, one village was randomly selected. People aged more than 18 years in the selected village were asked to participant in this study. In total, 879 participants were interviewed. The response rate was 94.9% (879/926).

### Data collection

The data for this study were collected in November 2019. All the interviewers were trained postgraduate students who understood the research and questionnaires. The participants were voluntary and provided written informed consent. For illiterate and semi-illiterate participants, written informed consent was filled by their legal guardians. The interviewers and subjects had face-to-face interviews, and the interviewers filled the questionnaires according to the subjects’ responses. After the survey was completed, at least two trained students checked the contents of the questionnaires, and questionnaires with the missing or unclear data were revisited and refilled.

### Measures

#### Child maltreatment

The Childhood Trauma Questionnaire-Short Form (CTQ-SF; [58]) is a 28-item self-report scale rated on a five-point Likert scale ranging from None = 1 to Always = 5. A sample item was as follows “I thought that my parents wished I was never born”. The final score was the sum of all item scores, with higher scores reflecting more frequent and severe of child maltreatment were experiences before the age of 16. The CTQ-SF includes the coherence and viability of the constructs [58]. In this study, internal consistency α = 0.87 for both males and females.

#### Depression

The Center for Epidemiologic Studies-Depression (CES-D; [59]) is a brief 20-item self-report measure (e.g., “I felt your life is failing” and “I felt lonely”) rated on a four-point Likert from 0 = Within 1 d to 3 = Five to Seven days. The final score was the sum of all items’ scores, with higher scores representing higher frequencies of depression during the past week. A scale is a valuable tool for studying the relationships between depression and other variables [59]. In this study, internal consistency was α = 0.90 for males and females.

#### Coping skill

The Simple Coping Style Questionnaire (SCSQ; [60]) is a 20-item scale rated on a four-point Likert scale ranging from Untaken = 0 to Often = 3 in the context of Chinese culture. The scale reflected that you possibly took actions or exhibited attitudes when you suffered setbacks and encountered difficulties. Items 1–12 belong to positive coping (e.g., “Tried to see the bright side of things”), and 13–20 belong to negative coping (e.g., “Tried to forget the whole thing”) [61]. The final score is the sum of all item scores, with the higher scores representing greater coping skills. The internal consistency coefficient of the scale was 0.90 [60]. In this study, internal consistency was α = 0.61 for males and females.

#### Loneliness

The Emotional and Social Loneliness Scale (ESLS; Wittenberg, 1986, cited in PR Shaver and KA Brennan [62]) is a 10-item rating on a five-point Likert ranging from 1 (never) to 5 (very often). A sample item is as follows: I haven’t special love relationship”. There are five items which are reverse scores (e.g., “Someone could accompany me”. The final score is the sum of all items’ scores, with higher scores reflecting a higher level of loneliness in the past year. For this study, internal consistency was α = 0.75 for both males and females.

#### Social-demographic variables

Gender was measured as male (1) and female (2). The participants’ ages were calculated using their date of birth and divided into three groups, 18–44 years old belong to young people (1), 45–64 years old belonging to middle-aged people (2), and above 65 years old belonging to old people (3). Ethnicity was assessed using the Hans (1) and others (2). Marital status was assessed as unmarried (1) and married (2). Education was assessed by illiteracy and semi-illiteracy (1), primary school (2) and middle school and above (3). Only child was assessed as yes (1) and no (2). Living alone was evaluated using yes (1) or no (2). Offspring were evaluated by yes (1) and no (2). Income level was assessed as higher (1), average (2) and lower (3).

#### Statistical methods

Statistical analyses were performed by using SPSS, version 23.0. Descriptive analyses were examined as means and standard deviations for continuous variables and numbers and percentages for categorical variables. One-way ANOVA or Chi-square test was conducted to assess mean differences for variables across the gender. Bi-correlation analysis was conducted with independent variables, mediators, and outcome variables. Linear regression was used to build the relationship between child maltreatment, loneliness, coping skills and depression while controlling for sociodemographic variables. Categorical variables are transformed into dummy variables. We conducted separate analyses of the data split by sex. All significance tests were two-tailed and a p-value of 0.05 or lower would be considered statistically significant.

## Results

### Descriptive statistic and Bivariate correlation

This study investigated 879 participants from rural communities in Shandong Province, China. The sample characteristics and descriptive analyses are revealed in Table 1. Most males (43.2%) were older, while most females (42.9%) were middle-aged. The percentage of married women was higher than that of married men (98.4% vs 96.3%, *p* < 0.05). Of the women, 44.9% were illiterate and semi-illiterate, and 45.7% males had a middle school education and above. Most participants weren’t only child, had offspring, and lived with at least one person, with percentages of 96.2%, 96.9%, and 89.3%, respectively. The mean CM score of the participants was 42.10 (SD = 0.90). Females were more depressed and lonelier than males, but there was not significant difference (*F* = 3.261, *p* > 0.05; *F* = 3.25, *p* > 0.05). More detailed information is provided in Table 1.

**Table 1:** Description and single analysis for association between sociodemographic characteristics, CM, loneliness, coping skills, and depression among male and females [Mean ± SD/N (%)]

Variables	Overall	Male	Female	F / χ^2^
Age				
18–44	205 (23.3)	60 (18.6)	145 (26.0)	14.32**
45–64	362 (41.2)	123 (38.2)	239 (42.9)	
≥ 65	312 (35.5)	139 (43.2)	173 (31.1)	
Ethnicity				
Hans	857 (97.5)	313 (97.2)	544 (97.7)	0.18
Others	22 (2.5)	9 (2.8)	13 (2.3)	
Married status				
Unmarried	21 (2.4)	12 (3.7)	9 (1.6)	3.90*
Married	858 (97.6)	310 (96.3)	548 (98.4)	
Education				
(Semi)-illiteracy	320 (36.4)	70 (21.7)	250 (44.9)	48.297***
Primary school	246 (28.0)	105 (32.6)	141 (25.3)	
Middle school and above	313 (35.6)	147 (45.7)	166 (29.8)	
Only child				
Yes	33 (3.8)	17 (5.3)	16 (2.9)	3.27
No	846 (96.2)	305 (94.7)	541 (97.1)	
Living alone				
Yes	94 (10.7)	34 (10.6)	60 (10.8)	0.01
No	785 (89.3)	288 (89.4)	497 (89.2)	
Offspring				
Yes	853 (96.9)	309 (96.0)	543 (97.5)	1.59
No	27 (3.1)	13 (4.0)	14 (2.5)	
Income level				
Higher	119 (13.5)	41 (12.7)	78 (14.0)	0.42
Average	563 (64.1)	206 (64.0)	357 (64.1)	
Lower	197 (22.4)	75 (23.3)	122 (21.9)	
CM	42.10 ± 10.77	43.05 ± 10.50	41.55 ± 10.89	3.99*
Loneliness	20.71 ± 7.12	20.14 ± 7.13	21.04 ± 7.10	3.25
Coping skills	32.40 ± 7.54	32.54 ± 7.58	32.32 ± 7.52	0.18
Depression	10.16 ± 10.14	9.34 ± 9.49	10.62 ± 10.48	3.261

*SD* denotes to standard deviation. Note: *, *p* < 0.05; **, *p* < 0.01; ***, *p* < 0.001. CM denotes to child maltreatment

Bivariate correlation analysis revealed that CM, loneliness, coping skills and depression had mutually have significant associations (*p* < 0.001), as shown in Table 2. Greater severity of CM was associated with fewer coping skills (*r* = -0.214, *p* < 0.001) and with more depression (*r* = 0.330, *p* < 0.001), and more loneliness (*r* = 0.308, *p* < 0.001) for males. More severe child maltreatment was associated with fewer coping skills (*r* = -0.251, *p* < 0.001) and with more depression (*r* = 0.314, *p* < 0.001) and loneliness (*r* = 0.454, *p* < 0.001) for females.

**Table 2:** Bi-correlations analysis between CM, loneliness, coping skills and depression for males and females

	1	2	3	4
1. CM		0.454***	-0.251***	0.314***
2. Loneliness	0.308***		-0.332***	0.568***
3. Coping skills	-0.214***	-0.314***		-0.316***
4. Depression	0.330***	-0.501***	-0.306***	

*CM* denotes to child maltreatment. Note: ***, *p* < 0.001. The upper diagonal represents the correlation for female, and the lower diagonal represents the correlation for male

### Mediation analysis for males

**Fig. 1:**
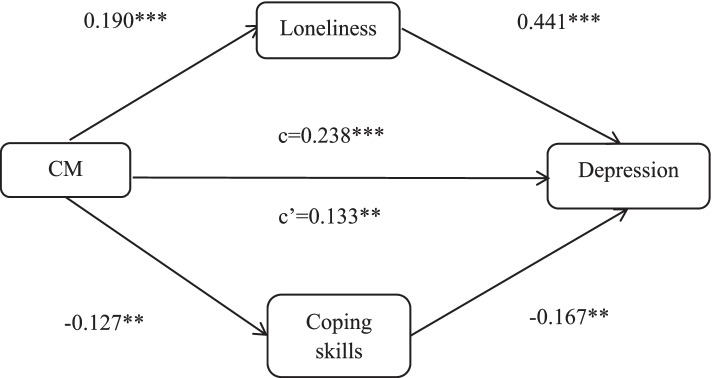
Regression coefficients for the relationship between CM and depression mediated by loneliness and coping skills among males. **, *p* < 0.01 ***, *p* < 0.001

A mediation model was used to examine the mediating role of loneliness and coping skills on the relationship between CM and depression. Table 3 demonstrates that males who experienced child maltreatment reported higher levels of loneliness (path a: b = 0.190, *p* < 0.001) and lower levels of coping skills (path a: b = -0.127, *p* < 0.01). The effects of loneliness and coping skills on depression were significant (path b: b = 0.441, *p* < 0.001; b = -0.167, *p* < 0.01). A total effect of child maltreatment on depression was observed (c = 0.238, *p* < 0.001). After controlling for mediative variables, the link between maltreatment and depression remained significant (direct effect c’: b = 0.133, *p* < 0.01). Tests of the indirect effect of loneliness (ab = 0.084) and coping skills (ab = 0.021) were significant. Figure 1 presents the mediating role of loneliness and coping skills in child maltreatment and depression among males. Loneliness and coping skills partially mediated the relationship between child maltreatment and depression among males.

**Table 3:** Mediation model: regression equation for CM, loneliness, coping skills and depression

				95%CI	
Path		B	Sig	LB	UB
Male					
a	CM → Loneliness	0.190	0.000	0.120	0.261
	→ Coping skills	-0.127	0.002	-0.205	-0.049
b	Loneliness → Depression	0.441	0.000	0.303	0.579
	Coping skills → Depression	-0.167	0.008	-0.291	-0.043
c	CM → Depression	0.238	0.000	0.147	0.330
c’	CM → Depression	0.133	0.003	0.045	0.222
Female					
a	CM → Loneliness	0.265	0.000	0.217	0.312
	→ Coping skills	-0.113	0.000	-0.166	-0.059
b	Loneliness → Depression	0.666	0.000	0.549	0.782
	Coping skills → Depression	-0.095	0.072	-0.199	0.008
c	CM → Depression	0.223	0.000	0.150	0.295
c’	CM → Depression	0.036	0.327	-0.036	0.107

Notes: Controlling for age, ethnicity, married status, education, only child, living alone, offspring and income level. *CM* denotes to child maltreatment, *CI* denotes to confidence interval, *LB* denotes to lower bound, *UB* denotes to upper bound

### Mediation analysis for females

**Fig. 2:**
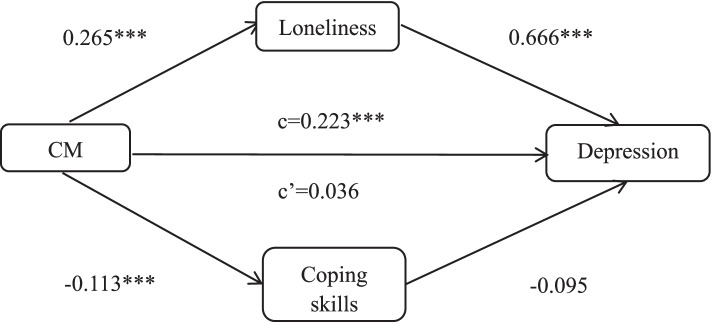
Regression coefficients for the relationship between CM and depression mediated by loneliness and coping skills among females. **, *p* < 0.01 ***, *p* < 0.001

Table 3 demonstrates that female participants who experienced child maltreatment reported higher levels of loneliness (path a: b = 0.265, *p* < 0.001) and lower levels of coping skills (path a: b = -0.113, *p* < 0.001). The effect of loneliness on depression was significant (path b: b = 0.666, *p* < 0.001), but the effect of coping skills on depression was not significant (path b: b = -0.095, *p* = 0.072). A total effect of child maltreatment on depression was observed (c = 0.223, *p* < 0.001). However, after controlling for mediative variables, the link between maltreatment and depression was not significant (c’: b = 0.036, *p* = 0.327). The indirect effects of loneliness (ab = 0.176) and coping skills (ab = 0.011) were significant. Figure 2 presents the mediating role of loneliness in child maltreatment and depression in females. Loneliness fully mediates the relationship between child maltreatment and depression in females. However, coping skills did not mediate the relationship between child maltreatment and depression among females.

## Discussion

This study used a population-based sample of rural participants and validated scales to examine the experiences of child maltreatment, loneliness, coping skills and depression. More importantly, we aimed to test the association between child maltreatment and depression, extend the roles of loneliness and coping skills in the relationship between child maltreatment and depression, and compare whether the mediation model for males differed from females.

In this study, males were more likely to be maltreated than females before 16 years of age, while the participants were from the rural regions. One possible reason may be that rural parents paid attention to boys, and they pinned greater hopes for boys, so boys were maltreated more than girls. The finding that nearly half of the females did not accept education also proved this phenomenon. As mentioned in the introduction section, we found an association between child maltreatment and depression in rural Chinese men and women.

This study supports the role of loneliness and coping skills in the relationship between child maltreatment and depression, which is consistent with previous research [20, 23]. The mediation models of loneliness and coping skills for men and women had similarities and differences. A previous study also indicated that sex differences mediated coping styles such as self-blame, fantasizing, problem avoidance, and rationalization on the relationship between childhood maltreatment and depressive symptoms [22]. The results proved that child maltreatment was directly predictive of increased loneliness and decreased coping skills; loneliness and low coping skills would worsen depressive symptoms. Loneliness and coping skills partially mediated the relationship between child maltreatment and depression for males. Loneliness fully mediated the relationship between child maltreatment and depression for females, but coping skills did not. For males, child maltreatment directly influenced depression, loneliness, and coping skills, while maltreatment indirectly caused depression because of loneliness and poor coping skill. Child maltreatment caused loneliness, thereby indirectly influencing depression in females.

Previous studies found that females were more prone to depression [10], and this study also found the average depressive level of females was higher than that of males, but there was no significant evidence to investigate this point (*F* = 3.261, *p* > 0.05), possibly because research methods, measures and participants were different from other studies. In this rural population, coping skills as a protective factor, and higher coping skills may decrease the influence of child maltreatment on depression. Furthermore, loneliness is a positive factor in people’s mental health. The findings regarding gender differences revealed loneliness played a more important role in the influence of child maltreatment on depression for females; depressive symptoms caused by child maltreatment were fully mediated and led by loneliness. And females were more likely to be influenced deeply by child maltreatment than males.

It is noteworthy that women were shown to be more vulnerable to loneliness, and society should provide more support and care for them, prevent and intervene in loneliness, and improve their coping skills. To summarize, we recommend that relevant departments promote education to increase individual quality, decrease the incidence of child maltreatment, and provide more social supports and assistance, consequently improving mental health.

This study has some limitations that must be considered. First, this was a cross-sectional study, and we measured independent, mediative, and dependent factors simultaneously. Hence, the child maltreatment and depression sequence were ambiguous, and perhaps child maltreatment influenced depression. It is also possible that depressive children were likely to be maltreated. Second, participants may have forgotten the maltreatment experience because of age, and some participants were shame to reflect on the experience. The study results were lower than expected; however, the outcomes were statistically significant. Third, we did not explore the different types of child maltreatment or the correlation between loneliness and coping skills, lonely people were less likely to develop the skills to cope with difficulties.

## Conclusions

In conclusion, males were more likely to experience child maltreatment than females. We also found an association between maltreatment and depression, the mediation models are different based on gender, the mediative role of loneliness and coping skills for males, and the mediative role of loneliness for females. The effects of loneliness on females who experienced maltreatment were greater than those of males who experienced maltreatment.

## Data Availability

Data are however available from the authors upon reasonable request and with permission of IRB of Shandong University School of public health, which were used under license for the current study, and so are not publicly available.

## References

[1] Afifi TO (2011). Child maltreatment in Canada: An understudied public health problem. Can J Public Health.

[2] Hengartner MP, Cohen LJ, Rodgers S, Muller M, Rossler W, Ajdacic-Gross V (2015). Association between childhood maltreatment and normal adult personality traits: exploration of an understudied field. J Pers Disord.

[3] Wan G, Tang S, Xu Y (2020). The prevalence, posttraumatic depression and risk factors of domestic child maltreatment in rural China: A gender analysis. Child Youth Serv Rev.

[4] Cui N, Xue J, Connolly CA, Liu J (2016). Does the gender of parent or child matter in child maltreatment in China?. Child Abuse Negl.

[5] Wan G, Pei T, He X, Zhang B: The Association Between Child Maltreatment and Family Structures: Evidence from Children in Rural China. J Fam Violence. 2022;37:1–13.

[6] Wan G, Li L, Gu Y (2021). A National Study on Child Abuse and Neglect in Rural China: Does Gender Matter?. Journal of family violence.

[7] Fang X, Fry DA, Ji K, Finkelhor D, Chen J, Lannen P, Dunne MP (2015). The burden of child maltreatment in China: a systematic review. Bull World Health Organ.

[8] Ren X, Yu S, Dong W, Yin P, Xu X, Zhou M (2020). Burden of depression in China, 1990–2017: findings from the global burden of disease study 2017. J Affect Disord.

[9] Li H, Ge S, Greene B, Dunbar-Jacob J (2019). Depression in the context of chronic diseases in the United States and China. International journal of nursing sciences.

[10] Zhang L, Xu Y, Nie H, Zhang Y, Wu Y (2012). The prevalence of depressive symptoms among the older in China: a meta-analysis. Int J Geriatr Psychiatry.

[11] Lei L, Mengyuan M, Hongye P, Zaofang Y, Miyuan W, Mengchao H, Zhenhai Z (2021). Prevalence and associated factors of depressive symptoms in China's rural elderly. Chinese General Practice.

[12] Vos T, Lim SS, Abbafati C, Abbas KM, Abbasi M, Abbasifard M, Abbasi-Kangevari M, Abbastabar H, Abd-Allah F, Abdelalim A (2020). Global burden of 369 diseases and injuries in 204 countries and territories, 1990–2019: a systematic analysis for the Global Burden of Disease Study 2019. The Lancet.

[13] Brown S, Fite PJ, Stone K, Bortolato M (2016). Accounting for the associations between child maltreatment and internalizing problems: The role of alexithymia. Child Abuse Negl.

[14] Gallo EAG, Munhoz TN (2018). Loret de Mola C, Murray J: **Gender differences in the effects of childhood maltreatment on adult depression and anxiety: A systematic review and meta-analysis**. Child Abuse Negl.

[15] VanMeter F, Handley ED, Cicchetti D (2020). The role of coping strategies in the pathway between child maltreatment and internalizing and externalizing behaviors. Child Abuse Neglect.

[16] Dumont M, Provost MA (1999). Resilience in adolescents: Protective role of social support, coping strategies, self-esteem, and social activities on experience of stress and depression. J Youth Adolesc.

[17] Kelly MM, Tyrka AR, Price LH, Carpenter LL (2008). Sex differences in the use of coping strategies: predictors of anxiety and depressive symptoms. Depress Anxiety.

[18] Milojevich HM, Levine LJ, Cathcart EJ, Quas JA (2018). The role of maltreatment in the development of coping strategies. J Appl Dev Psychol.

[19] Luo S, Liu Y, Zhang D (2020). Psychological maltreatment and loneliness in Chinese children: The role of perceived social support and self-esteem. Child Youth Serv Rev.

[20] Shevlin M, McElroy E, Murphy J (2015). Loneliness mediates the relationship between childhood trauma and adult psychopathology: evidence from the adult psychiatric morbidity survey. Soc Psychiatry Psychiatr Epidemiol.

[21] Hager AD, Runtz MG (2012). Physical and psychological maltreatment in childhood and later health problems in women: An exploratory investigation of the roles of perceived stress and coping strategies. Child Abuse Negl.

[22] Song X, Wang S, Wang R, Xu H, Jiang Z, Li S, Zhang S, Wan Y (2020). Mediating effects of specific types of coping styles on the relationship between childhood maltreatment and depressive symptoms among Chinese undergraduates: the role of sex. Int J Environ Res Public Health.

[23] Cantave CY, Langevin S, Marin M-F, Brendgen M, Lupien S, Ouellet-Morin I (2019). Impact of maltreatment on depressive symptoms in young male adults: The mediating and moderating role of cortisol stress response and coping strategies. Psychoneuroendocrinology.

[24] Perry BD: Child maltreatment: A neurodevelopmental perspective on the role of trauma and neglect in psychpathology. 2008.

[25] Lo CC, Cheng TC (2007). The impact of childhood maltreatment on young adults' substance abuse. Am J Drug Alcohol Abuse.

[26] Werner KB, McCutcheon VV, Challa M, Agrawal A, Lynskey MT, Conroy E, Statham DJ, Madden PAF, Henders AK, Todorov AA (2016). The association between childhood maltreatment, psychopathology, and adult sexual victimization in men and women: results from three independent samples. Psychol Med.

[27] Jaffee SR (2017). Child Maltreatment and Risk for Psychopathology in Childhood and Adulthood. Annu Rev Clin Psychol.

[28] Hamilton JL, Shapero BG, Stange JP, Hamlat EJ, Abramson LY, Alloy LB (2013). Emotional Maltreatment, Peer Victimization, and Depressive versus Anxiety Symptoms During Adolescence: Hopelessness as a Mediator. J Clin Child Adolesc Psychol.

[29] Jardim GBG, von Gunten A, da Silva Filho IG, Ziegelmann PK, Bumaguin DB, Nogueira EL, Engroff P, Neto AC (2019). Relationship between childhood maltreatment and geriatric depression: the mediator effect of personality traits. Int Psychogeriatr.

[30] Shahab MK, de Ridder JA, Spinhoven P, Penninx BWJH, Mook-Kanamori DO, Elzinga BM (2021). A tangled start: The link between childhood maltreatment, psychopathology, and relationships in adulthood. Child Abuse Negl.

[31] Bernet CZ, Stein MB (1999). Relationship of childhood maltreatment to the onset and course of major depression in adulthood. Depress Anxiety.

[32] Vranceanu A-M, Hobfoll SE, Johnson RJ (2007). Child multi-type maltreatment and associated depression and PTSD symptoms: the role of social support and stress. Child Abuse Negl.

[33] Kisely S, Abajobir AA, Mills R, Strathearn L, Clavarino A, Najman JM (2018). Child maltreatment and mental health problems in adulthood: birth cohort study. Br J Psychiatry.

[34] Novelo M, von Gunten A, Gomes Jardim GB, Spanemberg L, Argimon IIL, Nogueira EL (2018). Effects of childhood multiple maltreatment experiences on depression of socioeconomic disadvantaged elderly in Brazil. Child Abuse Negl.

[35] Arnow BA, Blasey CM, Hunkeler EM, Lee J, Hayward C (2011). Does gender moderate the relationship between childhood maltreatment and adult depression?. Child Maltreat.

[36] Scarpa A, Haden SC, Abercromby JM (2010). Pathways linking child physical abuse, depression, and aggressiveness across genders. Journal of Aggression, Maltreatment & Trauma.

[37] Pimlott-Kubiak S, Cortina LM (2003). Gender, victimization, and outcomes: reconceptualizing risk. J Consult Clin Psychol.

[38] Gover AR (2004). Childhood sexual abuse, gender, and depression among incarcerated youth. Int J Offender Ther Comp Criminol.

[39] Palgi Y, Shrira A, Ben-Ezra M, Shiovitz-Ezra S, Ayalon L (2012). Self- and other-oriented potential lifetime traumatic events as predictors of loneliness in the second half of life. Aging Ment Health.

[40] Lynch JJ, Convey WH (1979). Loneliness, disease, and death: Altemative approaches. Psychosomatics.

[41] Luo Y, Waite LJ (2014). Loneliness and mortality among older adults in China. J Gerontol B Psychol Sci Soc Sci.

[42] Cacioppo JT, Hughes ME, Waite LJ, Hawkley LC, Thisted RA (2006). Loneliness as a specific risk factor for depressive symptoms: cross-sectional and longitudinal analyses. Psychol Aging.

[43] Hawkley LC, Masi CM, Berry JD, Cacioppo JT (2006). Loneliness is a unique predictor of age-related differences in systolic blood pressure. Psychol Aging.

[44] Stein JY, Itzhaky L, Levi-Belz Y, Solomon Z (2017). Traumatization, loneliness, and suicidal ideation among former prisoners of war: a longitudinally assessed sequential mediation model. Front Psych.

[45] Gonzalez RM: Examining the relationship between loneliness and trauma through ecological momentary assessment. 2018.

[46] Arslan G, Yıldırım M: Psychological Maltreatment and Loneliness in Adolescents: Social Ostracism and Affective Experiences. Psychol Rep. 2021. 10.1177/00332941211040430 34396809

[47] Yan-Hui X, Rong Y, Jia-Xu D (2021). The Relationship between Childhood Maltreatment and Loneliness: The Mediating Roles of Rumination and Core Self-Evaluation. Journal of Psychological Science.

[48] Gibson RL, Hartshorne TS (1996). Childhood sexual abuse and adult loneliness and network orientation. Child Abuse Negl.

[49] Başoğlu E: The role of child abuse and gender on loneliness among university students. Middle East Technical University. 2019.

[50] Maloney E, Lapierre M, Cornetto K, Pears K (2006). Implications of neglect: An examination of underdeveloped social skills and loneliness in neglected children. Annual Meeting of the International Communication Association.

[51] Mikulincer M, Florian V, Weller A (1993). Attachment styles, coping strategies, and posttraumatic psychological distress: the impact of the Gulf War in Israel. J Pers Soc Psychol.

[52] Thabet AAM, Tischler V, Vostanis P (2004). Maltreatment and coping strategies among male adolescents living in the Gaza Strip. Child Abuse Negl.

[53] Perlman MR, Dawson AE, Dardis CM, Egan T, Anderson T (2016). The association between childhood maltreatment and coping strategies: The indirect effect through attachment. J Genet Psychol.

[54] Lepistö S, Paavilainen E (2012). The Association Between Child Maltreatment and Coping Strategies Among Finnish 9th Graders. Child Welfare.

[55] Hammen C (2005). Stress and depression. Annu Rev Clin Psychol.

[56] Bulletin of the Seventh National Census in China (NO. 3). http://www.stats.gov.cn/ztjc/zdtjgz/zgrkpc/dqcrkpc/. Accessed 3/1/2022.

[57] Shandong Statistic Yearbook. http://tjj.shandong.gov.cn/tjnj/nj2021/zk/indexch.htm. Accessed 3/1/2022.

[58] Bernstein DP, Stein JA, Newcomb MD, Walker E, Pogge D, Ahluvalia T, Stokes J, Handelsman L, Medrano M, Desmond D (2003). Development and validation of a brief screening version of the Childhood Trauma Questionnaire. Child Abuse Negl.

[59] Radloff LS (1977). The CES-D scale: A self-report depression scale for research in the general population. Appl Psychol Meas.

[60] Xie Y (1998). Preliminary study on the reliability and validity of the Simplified Coping Style Questionnaire. Chin J Clin Psychol.

[61] Zheng Z, Han W, Zhou Y, Zhang N (2020). Childhood Maltreatment and Depression in Adulthood in Chinese Female College Students: The Mediating Effect of Coping Style. Front Psych.

[62] Shaver PR, Brennan KA (1991). Measures of depression and loneliness. Meas Personal Soc Psychol Attitudes.

